# General Capacitance Upper Limit and Its Manifestation for Aqueous Graphene Interfaces

**DOI:** 10.3390/ijms241310861

**Published:** 2023-06-29

**Authors:** Alexey V. Butko, Vladimir Y. Butko, Yurii A. Kumzerov

**Affiliations:** Ioffe Institute, Polytechnicheskaya 26, 194021 St. Petersburg, Russia

**Keywords:** graphene, molecules, interface, nanostructures, capacitance, electric permittivity, capacitance limit, water, sensor, impedance spectroscopy

## Abstract

Double-layer capacitance (C_dl_) is essential for chemical and biological sensors and capacitor applications. The correct formula for C_dl_ is a controversial subject for practically useful graphene interfaces with water, aqueous solutions, and other liquids. We have developed a model of C_dl_, considering the capacitance of a charge accumulation layer (C_ca_) and capacitance (C_e_) of a capacitance-limiting edge region with negligible electric susceptibility and conductivity between this layer and the capacitor electrode. These capacitances are connected in series, and C_dl_ can be obtained from 1/C_dl_ = 1/C_ca_ + 1/C_e_. In the case of aqueous graphene interfaces, this model predicts that C_dl_ is significantly affected by C_e_. We have studied the graphene/water interface capacitance by low-frequency impedance spectroscopy. Comparison of the model predictions with the experimental results implies that the distance from charge carriers in graphene to the nearest molecular charges at the interface can be ~(0.05–0.1)nm and is about a typical length of the carbon-hydrogen bond. Generalization of this model, assuming that such an edge region between a conducting electrode and a charge accumulating region is intrinsic for a broad range of non-faradaic capacitors and cannot be thinner than an atomic size of ~0.05 nm, predicts a general capacitance upper limit of ~18 μF/cm^2^.

## 1. Introduction

To some degree, interfacial capacitance (C_int_) affects the functionality of almost all electronic devices, and understanding the fundamental principles and limitations of C_int_ is essential, particularly for chemical and biological sensors and capacitor applications. For example, graphene Solution Gated Field Effect Transistors (SGFETs) and other graphene-based devices are promising candidates for fabricating a new generation of effective chemical and biological sensors [1,2,3,4,5,6,7,8,9,10]. Sensor response in graphene SGFETs depends on charge accumulation in organic and hybrid nanostructures formed at the graphene interface with a solution-gating insulator. These nanostructures [5,11] are formed due to electrostatic interaction between charge carriers in graphene and molecular charges (ionic and dipole) driven by the applied gate voltage to the graphene interface from the gating solutions. The charge accumulation at this interface is strongly affected by C_int,_ which depends on the quantum capacitance of graphene (C_q_) and double-layer capacitance (C_dl_) [1,12,13,14,15,16,17]. 

Experimental studies by impedance spectroscopy of these capacitances were mainly fulfilled at the graphene interface with solutions of various salts. Meanwhile, the practically important interface between graphene and water is less studied experimentally. Moreover, among such reported investigations, there are few low-frequency measurements helpful in obtaining the electrostatic values of the capacitance that have been reported [18]. (We consider the frequency of the electric field to be low when its period provides a long enough charging time for the studied capacitor to get close to the fully charged state to obtain information on the upper limits of the capacitance). Partially for this reason, the correct formula and value of C_dl_ are controversial in cases of the graphene interface with water and molecular aqueous solutions. 

Several authors reported general theoretical models of the interfaces between conductors and electrolytes. Among the most prominent of these models is Parallel-Plate Condenser Helmholtz Model [19,20]. The main conclusion of this model is that the capacitance of an interface between a conductor and an electrolyte is primarily determined by a thin layer of molecular ions at this interface (Helmholtz layer) and that this capacitance can be estimated by using the formula for a planar parallel plate capacitor with a dielectric constant of this electrolyte [19,20]. Despite many theoretical advances, this conclusion, first suggested in 1853, is still a cornerstone of the presently used theories. Among the most important later-developed theories are Diffuse-Layer Model by Gouy and Chapman [19,20]. This model is based on the solution of the Poisson–Boltzmann equation that accounts for electric field screening by the electrolyte ions affected by their thermal motion and predicts the formation of a diffuse layer. A historical review of other important models can be found in [19,20]. For this paper, we summarize that in the presently used general theoretical models of the double-layer capacitance, the diffuse layer is positioned between the Helmholtz layer and the bulk of the electrolyte and capacitances of both layers are usually calculated by using two different dielectric constants for these layers [19]. The dielectric constant of the Helmholtz layer, that in some cases is divided by inner and outer Helmholtz planes, is usually considered to be smaller than the dielectric constant of the diffuse layer [21]. 

Significant progress was also achieved in simulation studies of specific types of interfaces that predict the dependences of relative electric permittivity (dielectric constant), electric potential, and field on the distance from the graphene/water interface (in some cases the dielectric constant equal to 1 was predicted at certain distances from the interface) [14,22,23,24,25]. Despite this progress in presently used theoretical calculations, the electric permittivity of the Helmholtz layer is often arbitrarily decreased from the bulk values to compensate for the overestimation of C_dl_. This overestimation occurs if the bulk values for this parameter are used [14,19]. Therefore, additional studies of double-layer capacitance formed at graphene/liquid interfaces are required. We focus this work on the low-frequency impedance study of graphene/water interfaces and on analysis of their capacitance characteristics which arise from the common non-faradaic capacitors’ structural features, to clarify the mentioned problems and to improve understanding of fundamental capacitance limitations. 

## 2. Results and Discussion

### 2.1. Model and Its Predictions

**Figure 1:**
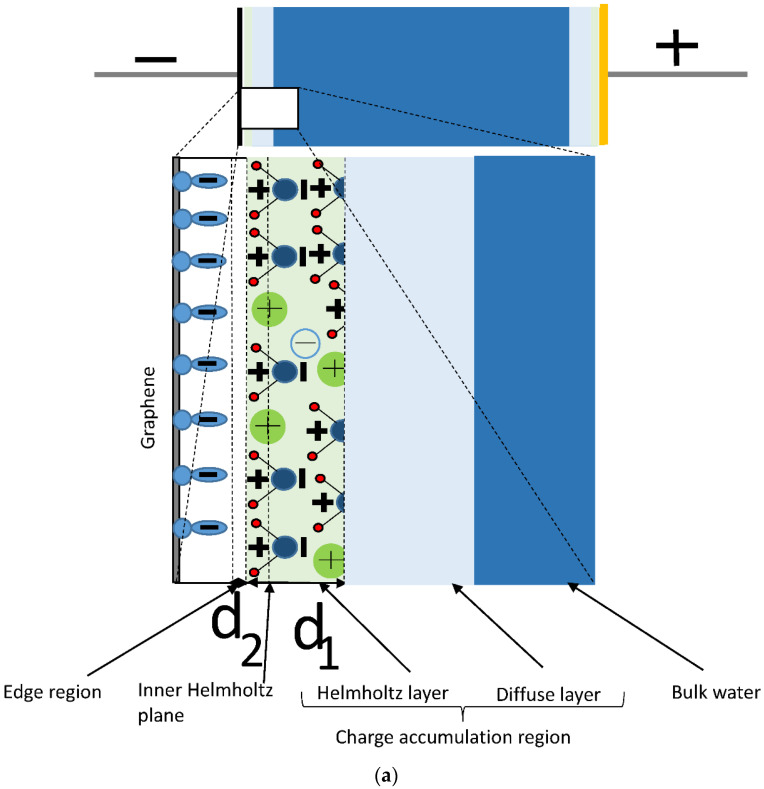

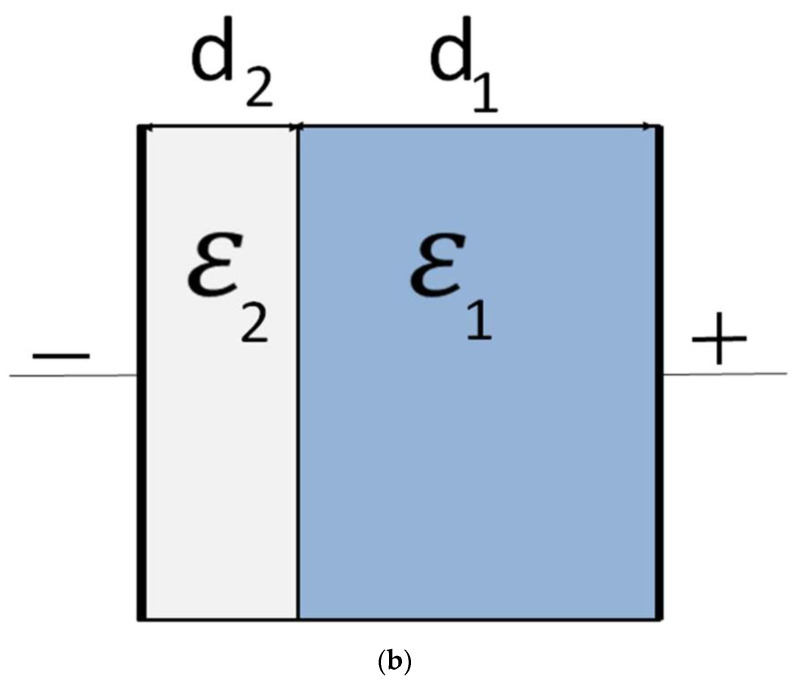
Schematic pictures: (**a**) A capacitor formed in water between graphene and gold gate electrode. The enlarged insert shows the graphene/water interface. Positive and negative molecular ions and molecular dipoles are shown in the water. π-orbital electrons are shown at the graphene surface. (**b**) A planar parallel plate capacitor containing two layers of different dielectric materials.

**Figure 2:**
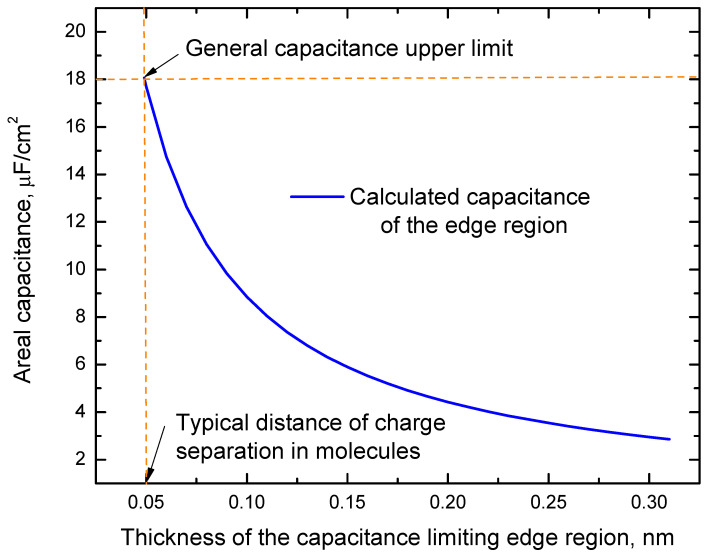
The dependence of C_e_ on the thickness of the edge layer (d_2_) is calculated by the formula C_e_ ≈ ε_0_/d_2_.

Several authors [12,19,20,21] estimate areal double layer capacitance at the interface between conducting electrode and electrolytes by using the formula for a planar parallel plate capacitor C_dl_ = ε_0_ε/t, where ε_0_ = 8.85 × 10^−12^ F/m, ε is the dielectric constant (relative permittivity) of the electrolyte, and t is the distance between plates. This formula assumes that edge effects at the interfaces of conducting electrodes and the dielectric material in capacitors can be neglected. This assumption is valid for most cases where capacitance values are not too high. However, for high enough capacitance, the edge effects related to an edge region without charge transfer and accumulation, with negligible conductivity, and electric susceptibility separating the conducting electrode from the charge accumulation region in non-faradaic capacitors may be necessary. This edge region can be empty or contain nanostructures other than polarized molecules or mobile charge carriers (ions or others). We will refer to this region as a capacitance-limiting edge or edge region. The existence of this region assumes that positive and negative charges at the interface between the electrode and charge accumulation region are separated by a distance that cannot be shorter than half of the typical length of the carbon-hydrogen bond and, in the absence of charge transfer through this interface in non-faradaic capacitors. Inside this region, charge accumulation does not occur, and the electric susceptibility is negligible. (According to the parallel plate capacitance model, the charge accumulation happens at the boundaries of this region). Figure 1a shows a schematic picture of a capacitor formed in water between graphene and gold gate electrode, illustrating this model in the graphene/water interface case. As one can see from this figure, the areal capacitances of the charge accumulation layer (C_ca_) and the edge region (C_e_) are connected in series and 1/C_dl_ = 1/C_ca_ + 1/C_e_. In theoretical studies, the inner Helmholtz plane (IHP) is often used [19,20,21]. This plane passes through the centers of the ions (see Figure 1a). We do not use this plane as a boundary of the edge region (see Figure 1a) because ions and dipoles have finite sizes. In our model, there must be no partial ionic or dipole charge density penetration inside the edge region to ensure that the electric susceptibility inside it is negligible. Dependence of C_e_ for the edge region with the relative permittivity, ε_2_ ≈ 1 on its thickness (d_2_) (see Figure 1a,b) is calculated by the parallel plate capacitor formula, C_e_ ≈ ε_0_/d_2_ (see the result of these calculations in Figure 2). Considering that d_2_ can unlikely be shorter than half of the typical length of a carbon-hydrogen bond (about 0.05 nm) from Figure 2, one can see that this smallest possible thickness of the edge layer corresponds to C_e_ ≈ 18 µF/cm^2^. The capacitance of the edge region is connected in series with other capacitances that determine C_dl,_ and this value of C_e_ can be used as an upper limit for C_dl_ reached at d_2_ ≈ 0.05 nm.

**Figure 3:**
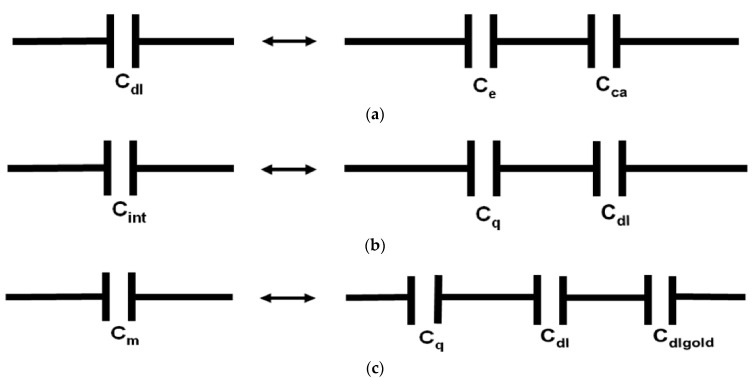
Schematic representation of (**a**) double layer capacitance (C_dl_) as the capacitance of edge region (C_e_) and capacitance of charge accumulation layer (C_ca_) connected in series, (**b**) Interfacial capacitance (C_int_) as the quantum capacitance of graphene (C_q_) and (C_dl_) connected in series, and (**c**) measured capacitance between the gate electrode and graphene as C_q_, C_dl_, and C_dlgold_ connected in series.

In considering interfaces between conductors and electrolytes, the charge accumulation layer is divided into two layers (Helmholtz layer and diffuse layer) [19,20,21]. In the Helmholtz layer, as usually defined in the literature, an edge region with a dielectric constant equal to 1 is not specified. By excluding a separate edge region from the Helmholtz layer, we are changing it. However, we will still refer to the leftover layer as the Helmholtz layer. Further analysis of C_dl_ can be done with the often-made assumption that the diffuse layer effect on C_dl_ can be neglected [19]. In this case, we obtain 1/C_dl_ = 1/C_e_ + 1/C_H_ (see Figure 3a), where C_H_ is the areal capacitance of the Helmholtz layer. This formula can be estimated by C_dl_ expression derived for a planar parallel plate capacitor containing two layers of different dielectric materials (see Figure 1b), C_dl_ = (ε_0_ε_1_ε_2_)/(d_1_ε_2_ + d_2_ε_1_), where ε_1_, ε_2_, d_1_, and d_2_ are the relative permittivities of the first and the second dielectric material, and thicknesses of the first and the second layer, respectively. In our model, the first and the second layers correspond to the Helmholtz layer and the edge region, respectively (see Figure 1a,b). The edge region has ε_2_ ≈ 1 and we obtain C_dl_ ≈ (ε_0_ ε_1_)/(d_1_ + d_2_ε_1_). One can also see in Figure 1a,b that the Helmholtz layer with thickness d_1_ in the water contains electrical poles of the polarized molecular dipoles and molecular ions of primarily the same sign. The correct calculation of ε_1_ is difficult, and most presently used models made arbitrary assumptions about ε_1_ and d_1_ to fit the experimental data best [19,21]. The typically used values for d_1_ are in the range (0.3–0.5) nm, and for ε_1_ are in the range (6–10) [19,20,21]. Excluding the edge region from the Helmholtz layer in this model may affect these estimates in a way that increases the capacitance of the Helmholtz layer to keep calculated C_dl_ values in agreement with the experimental data. We will analyze our experimental data to obtain more specific information about how introducing the edge region affects the Helmholtz layer parameters.

Generalization of this model, assuming that a capacitance limiting edge region between a conducting electrode and charge accumulating region is intrinsic in a broad range of non-faradaic capacitors and cannot be thinner than the typical distance between negative and positive charges in atoms of ~0.05 nm, predicts a general capacitance upper limit of ~18 µF/cm^2^. To obtain this limit, we consider that the capacitance (C_m_) of a capacitor measured between its electrodes cannot be larger than the capacitance of the edge region (C_e_) because it is connected in series with other capacitances in the capacitor schema. In an important example of a capacitor electrode formed by graphene atomic layers with a theoretical specific surface area of 2630 m^2^/g [26], the obtained general areal capacitance upper limit corresponds to the capacitance upper limit of about 473 F/g. 

Most of the experimental data on areal capacitances reported in the literature do not exceed the obtained general limit. Some authors [27] reported the areal capacitance values for non-faradaic capacitors that exceed this limit, usually by a factor of ~3 or less. These high reported values of the areal capacitances are probably obtained due to the normalization of the measured capacitances by the nominal surface areas of the capacitor electrodes in these papers. For correct interpretation of these data, one needs to remember that the nominal surface areas of the capacitor electrodes, in many cases, are a few times less than the true surface areas of the capacitor electrodes due to the electrode roughness or porosity. Therefore, the mentioned data do not contradict the existence of the general capacitance upper limit. This limit is universally applicable for non-faradaic capacitances. It may play a role for many faradaic capacitors as well. To determine the applicability of the discussed capacitance limit to different types of faradaic capacitors, detailed specific considerations are required.

### 2.2. Impedance Spectroscopy Results and Discussion

**Figure 4:**
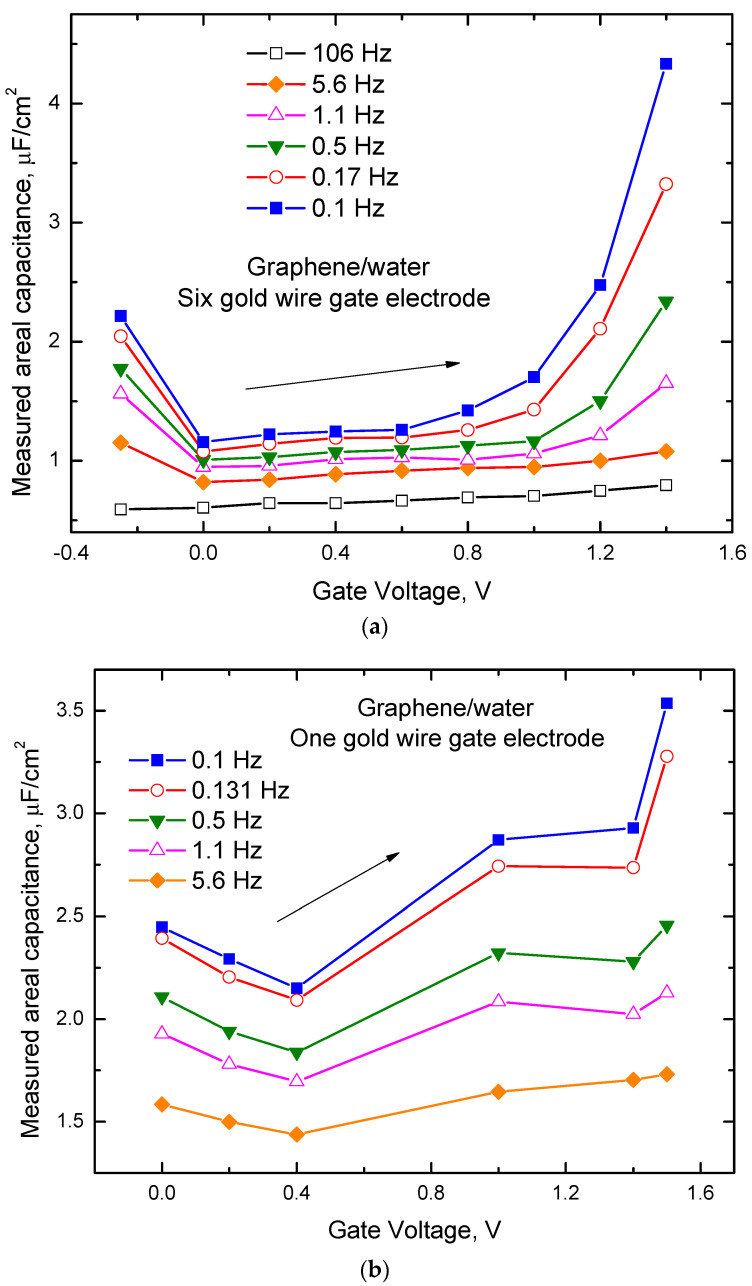
The capacitance normalized by graphene surface area measured in water with gate voltage applied at different frequencies between graphene and (**a**) six gold wire gate electrode and (**b**) one gold wire gate electrode. Directions of the arrows correspond to the measurement orders.

The interfacial capacitance at the graphene/water interface (C_int_) is determined by connected in series C_dl_ and C_q_ (see Figure 3b) [1,12,13,14,15,16,17], where, C_int_, C_dl_, and C_q_ are normalized by graphene electrode surface area (S_graphene_). The capacitance measured between graphene and the gold gate electrode is determined by connected in series C_int_ and C_dlgold_ (see Figure 3c), where C_dlgold_ is the double layer capacitance at the interface between water and gold gate electrode normalized by the gold gate electrode true surface area (S_gold_). Figure 4a,b shows the typical capacitances (C_m_) measured at different frequencies between graphene and two different types of gate electrodes in water. We are primarily interested in electrostatic values of the capacitances, and therefore, we focus our analysis on the experimental data obtained at the lowest frequencies (0.1 Hz). One can see from Figure 4a,b that at these frequencies for both types of gate electrodes, capacitance dependence on the gate voltage demonstrates a minimum. We identify the observed minimum in capacitance as corresponding to the Dirac point, similar to the conclusions of the work [12]. For both types of gate electrodes, capacitance increases with the gate voltage shifting away from the Dirac point. Considering known C_q_ dependence on the gate voltage [12] and assuming the usually weak dependence of the double-layer capacitance on the gate voltage, we conclude that in the studied gate voltage range C_q_ plays a significant role in the interfacial capacitance for both types of the used gate electrodes. One can also see from Figure 4a,b that the dependence of C_m_ on the gate voltage is stronger for the gate electrode with a higher surface area. This behavior can be explained by the fact that for the gold gate electrode with higher surface area C_dlgold_ is also higher in comparison with C_q_, and because these capacitances connected in series C_q_ for this type of gate electrode plays a more critical role in the dependence of C_m_ on the gate voltage.

**Figure 5:**
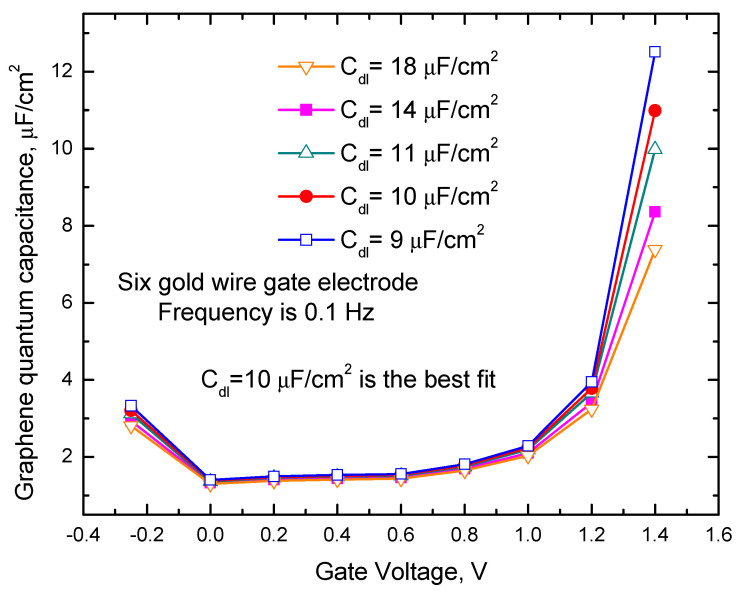
Graphene quantum capacitances were obtained using five different C_dl_ values, and the capacitance was measured between six gold wire gate electrodes and graphene in water at a frequency of 0.1 Hz.

From Figure 4a one can also see that C_m_ slope versus gate voltage increase with the gate voltage increase. The maximal slope of the measured capacitance versus gate voltage is about 10 µF/(cm^2^V). This slope is also about two times less than the slope predicted for quantum capacitance by the formula C_q_ ≈ (2 e^3^ V_ch_)/(π ћ^2^ ν_F_^2^) [12], where ћ is Planck constant, e is an electron charge, ν_F_ ≈ c/300 is the Fermi velocity of the Dirac electrons, and V_ch_ is the potential of graphene. Considering schematic pictures in Figure 3a–c quantum capacitance value can be calculated from the equation 1/(C_m_ S_graphene_) = 1/(C_q_ S_graphene_) + 1/(C_dl_ S_graphene_) + 1/(C_dlgold_ S_gold_). Results of these calculations for the sample with the gate electrode containing six gold wires (see the data presented in Figure 4a) with different C_dl_ values are shown in Figure 5. In these calculations, it was assumed that the roughness of our gold gate electrodes and the electrode of the capacitors studied in [27] are about the same and using their capacitance measurement results, we take C_dlgold_S_gold_ ≈ 40 µF/cm^2^ S_goldnom_, where_._ 40 µF/cm^2^ is the average capacitance value reported for gold electrodes at 0.1 Hz frequency in [27], and S_goldnom_ is the nominal surface area of the capacitor electrode.

We also assume that the roughness of graphene is small and that the true and the nominal surface areas of graphene electrodes are equal. Figure 5 shows that the obtained quantum capacitance data qualitatively are similar to the data reported in the paper [12]. The results shown in Figure 5 at 1.4 V are compared with theoretical predictions for the quantum capacitance values calculated by the mentioned above formula [12]. In this comparison, C_dl_ is used as an adjustable parameter. The best fit is obtained at C_dl_ ≈ 10 µF/cm^2^. A similar analysis based on the data presented in Figure 4b at the gate voltage of 1.4 V for the sample with one gold wire gate electrode gives the best fit with C_dl_ ≈ 12 µF/cm^2^. We need to clarify that the calculations of C_dl_ made in this paper are based on a few assumptions and should be considered simple estimates. Therefore, based on our results, we can only conclude that C_dl_ is significantly affected by C_e_ and that C_dl_ is approaching the range that is close to the general capacitance upper limit of ~18 µF/cm^2^ for the graphene/water interface rather than speculate about the exact values of C_dl_. The obtained results are in reasonable agreement with the estimates of C_dl_ ≈ (10–20) µF/cm^2^ that were based on the capacitance measurements at the interfaces of graphite with aqueous solution [12]. The values for C_dl_ obtained for the gate voltage of 1.4 V are significantly larger than estimates of C_dl_ ≈ 2–3 µF/cm^2^ obtained from the simulations based on a 0.3 nm thick hydrophobic gap at the water/graphene interface in work [22]. 

In the case of C_e_ << C_H_, we obtain C_e_ ≈ C_dl._ Considering that C_e_ ≈ ε_0_/d_2_ for C_dl_ ≈ 10 µF/cm^2^ we get d_2_ ≈ 0.09 nm. The C_dl_ value decrease of C_H_ (see Figure 3a) can only increase C_e_, making d_2_ smaller. Comparison of our model predictions with our capacitance measurement results provides estimates of the distances separating π-orbital charge carries in graphene and molecular charges at the interface between graphene and water as (0.05–0.09) nm. The obtained distances are comparable with typical lengths separating positive and negative charges in molecular compounds. For C_dl_ ≈ 10 µF/cm^2^, considering the upper limit for C_e_ ≈ 18 µF/cm^2^, the smallest possible value of C_H_ ≥ 22.5 µF/cm^2^ can be calculated from the formula 1/C_H_ ≈ 1/C_dl_ − 1/C_e_. Therefore, the estimate of C_H_ ≈ 18 µF/cm^2^ [19] that was used before excluding the edge region from the Helmholtz layer likely needs to be increased.

Due to the existence of the edge region dependence of C_dl_ on the radius of the counter-ions that was often assumed for interfaces between conducting electrodes and electrolytes [12,19] needs to be significantly modified. Therefore, predictions of this model improve understanding C_dl_ dependent sensor selectivity for the detection of molecules in analyzed solutions.

## 3. Materials and Methods

In this work, we have studied the capacitance of the graphene/water interface by low-frequency impedance spectroscopy. We have used monolayer graphene fabricated by SiC thermal decomposition in our work [5]. All details of the graphene growth and the structural characterization were reported in that paper. We use silver paste contacts on the graphene surface to prevent possible damage to graphene from the alternative lithographic process of electrode preparation. Epoxy glue was used to cover the silver paste contact pads to prevent degradation of the electrodes during the measurements. These contacts on graphene were typically subject to temperature treatment at 80–100 °C for up to 2 h in a dry box. The typical distance between electrodes, their length, and their typical width is between (1–1.5) mm, (3–4 mm), and (2.5–3) mm, respectively. By comparing 2-contact and four-contact measurements and from the observed approximately linear scaling of the graphene resistance with the length of the graphene sample, we conclude that the contacts to graphene are ohmic. In this work, we explored non-faradaic capacitors with blocking polarizable electrodes that carbon (graphene) and gold form at the interface with water [19]. We use gold wires of 50 µm diameter positioned ~200 µm away from graphene as the gating electrodes. Two types of these gating electrodes have been used. One of these types was made with a single gold wire, and another with six gold wires.

Deionized water was placed between the gating electrode and graphene for electrical impedance spectroscopy (EIS) measurements. Nonpolarizable reference electrodes such as Ag/AgCl were not used to avoid the addition of Cl^−^ and other ions to the deionized water. The EIS measurements were performed with a potentiostat- galvanostat, Electrochemical Instruments P-45X, containing an FRA-24M module for measuring electrochemical impedance. Impedance was measured between the gate electrode and graphene for different applied gate voltages ranging from −0.2 V to 1.5 V with a DC offset scan within the amplitude range of ±20 mV in the frequency range from 0.1 to 106 Hz. This asymmetric voltage range was chosen to minimize possible hysteretic phenomena.

It should be noted that the theoretical activation potential of water decomposition is around 1.23 V. Still, experimentally, it has been shown [28] that the overpotential of the reaction is higher. Our work’s gate voltage and other conditions would not lead to any significant reaction. Due to the mentioned blocking nature of the contacts between water and electrodes, the capacitance (C_m_) between the gate electrodes and graphene can be obtained from the formula Z = 1/(i2πfC_m_), where f is the frequency of the applied gate voltage, i is the square root of negative 1, and Z is the measured impedance.

## 4. Conclusions

We have developed a model of C_dl_, considering the capacitance of a charge accumulation layer (C_ca_) and capacitance (C_e_) of an edge region with negligible electric susceptibility and conductivity that exists between this layer and the conducting electrode of a non-faradaic capacitor. These capacitances are connected in series, and C_dl_ can be obtained from 1/C_dl_ = 1/C_ca_ + 1/C_e_. In the case of the graphene/water interface, this model predicts that C_dl_ is significantly affected by C_e_. We have studied the capacitance of the graphene/water interface by low-frequency impedance spectroscopy. Comparison of the model predictions with the capacitance measurement results provides estimates of the distances separating π-orbital charge carriers in graphene and molecular charges at the interface between graphene and water of ~(0.05–0.09) nm. The obtained distances are comparable with typical lengths separating positive and negative charges in molecular compounds. Predictions of this model improve understanding of sensor selectivity for the detection of molecules. Generalization of this model, assuming that such an edge region between a conducting electrode and a charge accumulating region is intrinsic for a broad range of non-faradaic capacitors and cannot be thinner than an atomic size of ~0.05 nm, predicts a general capacitance upper limit of ~18 µF/cm^2^. 

## Data Availability

The data presented in this study are available on request from the corresponding author.
